# Long non-coding RNA MALAT1 for promoting metastasis and proliferation by acting as a ceRNA of miR-144-3p in osteosarcoma cells

**DOI:** 10.18632/oncotarget.19727

**Published:** 2017-07-31

**Authors:** Yong Wang, Yueyang Zhang, Tao Yang, Wei Zhao, Ningning Wang, Pengcheng Li, Xiandong Zeng, Weiguo Zhang

**Affiliations:** ^1^ The 4th Department of Orthopedic Surgery, Central Hospital Affiliated to Shenyang Medical College, Shenyang, P. R. China; ^2^ Department of Pathology, Liaoning Cancer Hospital & Institute, Shenyang, P. R. China; ^3^ Department of Joint Surgery, The First Affiliated Hospital of Dalian Medical University, Dalian, P. R. China; ^4^ The 2nd Department of Cardiology, Central Hospital Affiliated to Shenyang Medical College, Shenyang, P. R. China; ^5^ Department of Surgical Oncology, Central Hospital Affiliated to Shenyang Medical College, Shenyang, P. R. China

**Keywords:** lncRNA MALAT1, miR-144-3p, ceRNA, metastasis and proliferation, osteosarcoma

## Abstract

Long non-coding RNAs (lncRNAs) are involved in various biological processes and diseases including osteosarcoma. Long non-coding RNA metastasis-associated lung adenocarcinoma transcript 1 (MALAT1) is overly expressed in osteosarcoma. But the function and mechanism it works on in osteosarcoma proliferation and metastasis mediated by Rho associated coiled-coil containing protein kinase 1 (ROCK1) and Rho associated coiled-coil containing protein kinase 2 (ROCK2) remain unclear. In the present study, an elevated MALAT1 was found in osteosarcoma tissues and cell lines, and the elevated MALAT1 was correlated with a poor prognosis in osteosarcoma patients. The functional experiments show that a decreased MALAT1 could remarkably inhibit osteosarcoma cell metastasis and proliferation but induce cell cycle arrest, indicating that MALAT1 functioned as an oncogene in osteosarcoma. Furthermore, we confirmed that MALAT1 and ROCK1/ROCK2 which were targeted by microRNA-144-3p (miR-144-3p) shared the same miR-144-3p combining site. Furthermore, the constructed luciferase assay verified that MALAT1 was a target of miR-144-3p. Additionally, the results of a qRT-PCR demonstrated that MALAT1 and miR-144-3p repressed each other's expression in a reciprocal manner. Finally, we affirmed that an overexpression of MALAT1 inhibited ROCK1/ROCK2 expression and its mediated metastasis and proliferation by working as a competitive endogenous RNA (ceRNA) via miR-144-3p.

In summary, the findings of this study based on the ceRNA theory, combining the research foundation of miR-144-3p, ROCK1 and ROCK2, taking MALAT1 as a new point of study, provided new insights into molecular level proliferation reversal and metastasis of osteosarcoma.

## INTRODUCTION

As the most prevalent primary malignant tumor in adolescents, osteosarcoma is always regarded as one of the main reasons for cancer-related deaths in children and young adolescents [1–3]. A combination of surgical resection and chemotherapy for osteosarcoma has significantly improved the survival rate of affected individuals [4]. However, 30 - 40% of patients with localized osteosarcoma develop recurrent or metastatic diseases, and in such cases, the average survival period will be reduced to shorter than 1 year [5, 6]. Therefore, seeking out available metastasis and proliferation-related molecules and identifying their working mechanism appears to be especially urgent for both clinical surgeons and basic medical researchers.

Recently, long non-coding RNAs (lncRNAs), which are a group of RNAs that measure more than 200 nucleotides in length, have become the focus in numerous types of cellular processes including the modulation of cell proliferation, apoptosis and invasion, the reprogramming of stem cell differentiation and chromatin remodeling [7–10]. The metastasis-associated lung adenocarcinoma transcript 1 (MALAT1), also referred to as nuclearenriched abundant transcript 2 (NEAT2), is one of the first discovered cancer-associated lncRNAs [11–13]. It is an evolutionarily highly conserved and widely expressed long noncoding transcript with a length of 8,000 nucleotides. Deregulation or a functional role for MALAT1 has now been established in several solid tumors, including lung cancer, breast cancer, hepatocellular carcinoma, prostate cancer and gallbladder cancer [14–17]. However, the functional roles of MALAT1 in the tumorigenesis and progression of osteosarcoma have not been extensively studied.

MicroRNAs (miRNAs) are another type of noncoding RNA which are 20-200 nucleotides in length, and miRNAs are certified as an oncogene or tumor suppressor gene in multiple cancers via regulating its target genes by interfering with transcription or inhibiting translation [18, 19]. In the former research, microRNA-144 (miR-144) was reported as a tumor suppressor gene in osteosarcoma tissues and cell lines. Up-regulation of miR-144 could suppress osteosarcoma cells migration and invasion by targeting regulation of rho associated coiled-coil kinase 1 (ROCK1) and rho associated coiled-coil kinase 2 (ROCK2) [20]. The question arose and remains unclear, were there any molecules that could regulate miR-144 and its downstream ROCK1/ROCK2. Lately, the competitive endogenous RNAs (ceRNA) theory between miRNAs and lncRNAs is widely studied and currently prevalent. Luan W and workmates reported that MALAT1 promoted malignant melanoma proliferation and metastasis by acting as a ceRNA of miR-22 [21]. Cai X found that MALAT1 was up-regulated and functioned as an oncogene in osteosarcoma via RhoA and its downstream ROCKs [22]. To date, whether MALAT1 could affect ROCK1/ROCK2 via a ceRNA of miR-144 remains unclear.

Here, we clarified that MALAT1 was highly expressed in osteosarcoma and that down-regulation of MALAT1 decreased migration/invasion and proliferation in osteosarcoma cells MNNG/HOS. Meanwhile, we affirmed that MALAT1 was a target of miR-144-3p via the same binding sites as ROCK1/ROCK2. Also, we confirmed that MALAT1 could regulate ROCK1/ROCK2 and their mediated metastasis and proliferation by working as a ceRNA mechanism via miR-144-3p. Finally, through the use of murine models, we confirmed that MALAT1 worked in a facilitative role on osteosarcoma tumorigenesis and metastasis. The findings of the present study indicated that MALAT1 might be a new target in the molecular therapeutic of osteosarcoma.

## RESULTS

### Elevated MALAT1 was expressed and correlated with poor prognosis in osteosarcoma patients

**Figure 1:**
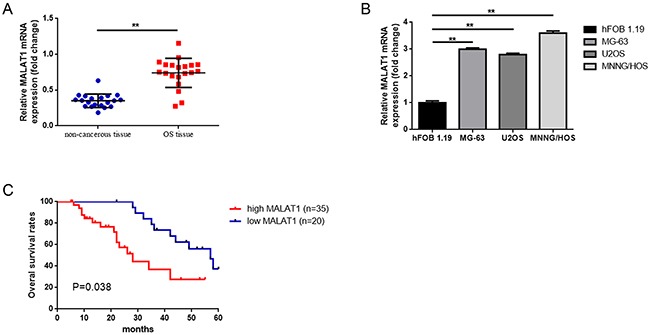
Elevated MALAT1 was expressed and correlated with poor prognosis in osteosarcoma patients **(A)** qRT-PCR was conducted to quantify the expression of MALAT1 in osteosarcoma tissues and paired noncancerous bone tissues (n = 55), using GAPDH as the normalization control. **P < 0.01 vs. noncancerous bone tissues group. **(B)** Expression MALAT1 in hFOB1.19, MG-63, U2OS and MNNG/HOS was determined by qRT-PCR, using GAPDH as the normalization control. **P < 0.01 vs. hFOB1.19. **(C)** The correlation between MALAT1 expression level and overall survival of patients with osteosarcoma was analyzed by Kaplan-Meier analysis (the log-rank test was used to calculate P-values). *P = 0.038 < 0.05 vs. low MALAT1 expression group. Data are shown as mean ± SD from three independent experiments.

Firstly, we detected the expression of MALAT1 in 20 osteosarcoma tissues and paired para-tumor bone tissues by using real-time quantitative PCR (qRT-PCR). Also, we measured MALAT1 expression in osteosarcoma cell lines MG-63, U2OS, MNNG/HOS and a human osteoblast cell line hFOB 1.19 via the same method. As the data presented in Figure 1A, 1B, the expression level of MALAT1 was remarkably elevated in osteosarcoma tissues and cell lines compared to para-tumor bone tissues and hFOB 1.19. Secondly, we tried to assess whether the elevated MALAT1 was correlated with the final survival time of osteosarcoma by using qRT-PCR. Our Kaplan–Meier analysis and log-rank test revealed that a high expression of MALAT1 was inversely correlated with osteosarcoma patients’ overall survival (Figure 1C). Thirdly, a further correlation analysis confirmed that the high level of MALAT1 was closely correlated with clinicopathological features, especially with regard to clinical stage, distant metastasis and tumor size (determined by using qRT-PCR) (Table 1).

**Table 1:** Association of MALAT1 expression with clinicopathological features of osteosarcoma

Features	No. of cases	MALAT1		P value †
		**High**	**Low**	
Age at diagnosis				0.786
<18	29	19	10	
≥18	26	16	10	
Gender				0.415
Female	29	20	9	
Male	26	15	11	
Histological subtype				0.732
Osteoblastic	9	5	4	
Chondroblastic	12	8	4	
Fibroblastic	11	6	5	
Mixed	13	7	6	
Clinical stage				0.05
I+IIA	21	10	11	
IIB/III	34	25	4	
Distant metastasis				0.01
Absent	24	9	15	
Present	31	26	5	
Tumor size (cm)				0.011
<5	25	11	14	
≥5	30	24	6	
Anatomic location				0.582
Tibia/femur	27	16	11	
Elsewhere	28	19	9	

† P-value obtained from Pearson chi-square test or Fisher's exact test.

### Down-regulation of MALAT1 inhibited migration/invasion and decreased ROCK1/ROCK2 expression in osteosarcoma cells MNNG/HOS

**Figure 2:**
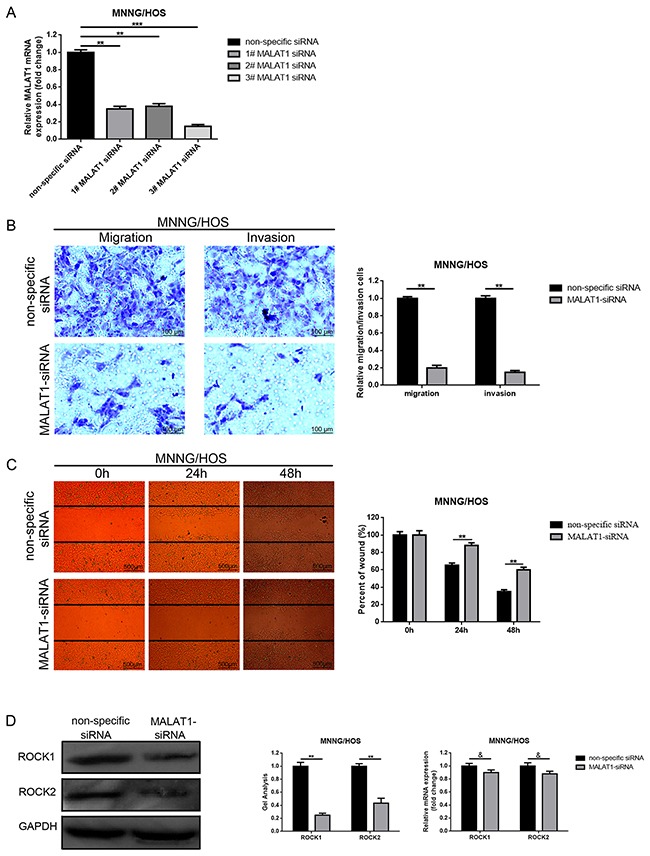
Depression of MALAT1 inhibited migration/invasion and decreased ROCK1/ROCK2 expression in osteosarcoma cells MNNG/HOS **(A)** The MALAT1 mRNA expression in MNNG/HOS cells after MALAT1 siRNA was determined by qRT-PCR. **(B** and **C)** The metastasis ability changes in MNNG/HOS cells after depression of MALAT1 were detected using transwell assays (B, scale bars 100 μm, magnifications, 10×) and wound healing assays (C, scale bars 500 μm, magnifications, 4×). **(D)** ROCK1 and ROCK2 expression in MNNG/HOS cells after depression of MALAT1 was measured by western blot and qRT-PCR. &P > 0.05, **P < 0.01, ***P < 0.001 vs. non-specific siRNA group. Data are shown as mean ± SD from three independent experiments.

Since elevated MALAT1 was closely related to distant metastasis in osteosarcoma patients, we tried to explore the potential function that MALAT1 may work on migration and invasion in the osteosarcoma cell line MNNG/HOS. Firstly, we created a loss of function cell model by transfection of MALAT1 siRNA. As shown in Figure 2A, among 1-3 # MALAT1-siRNAs, 3# MALAT1-siRNA presented the most effective decrease of effects. So, 3# MALAT1-siRNA was selected for the following RNAi experiments. Secondly, transwell assays and wound healing assays were executed to evaluate the ability to change metastasis. As displayed in Figure 2B, 2C, the migration and invasion ability of MNNG/HOS were notably weakened by transfection of MALAT1-siRNA compared to non-specific siRNA transfection.

In the former study, ROCK1/ROCK2 was reported as a metastasis-related gene in osteosarcoma [20, 23]. Hence, we also detected the effect on how MALAT1 works on ROCK1/ROCK2 expression. As presented in Figure 2D, the decrease of MALAT1 could reduce ROCK1/ ROCK2 at the post-transcriptional level.

### Down-regulation of MALAT1 inhibited cell proliferation while induced cell cycle arrest in MNNG/HOS cells MNNG/HOS

**Figure 3:**
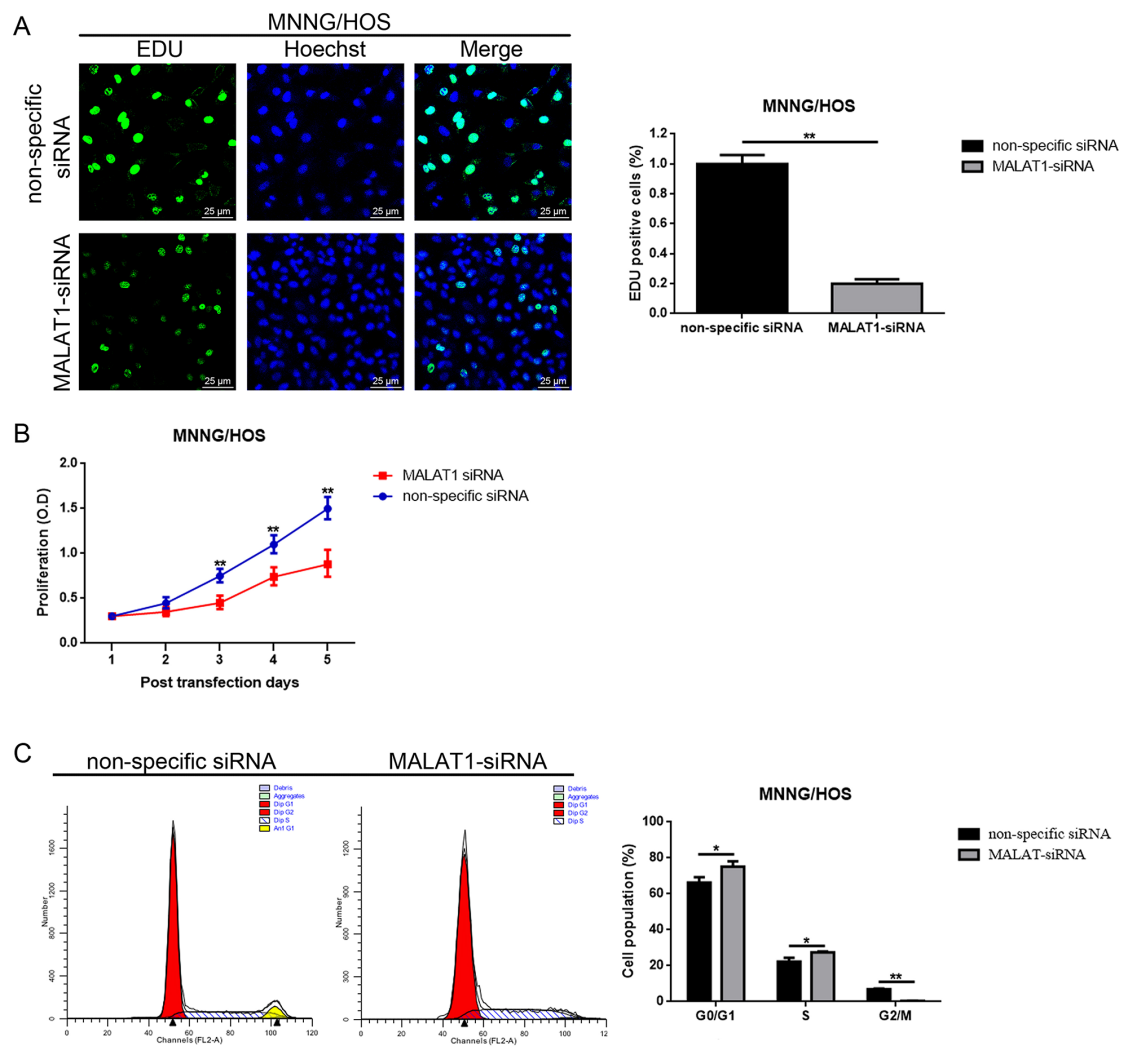
Down-regulation of MALAT1 inhibited cell proliferation while induced cell cycle arrest in MNNG/HOS cells MNNG/HOS **(A** and **B)** The proliferation ability changes of MNNG/HOS cells after down-regulation of MALAT1 were detected by 5-ethynyl-2’-deoxyuridine (EDU) incorporation assays (A, scale bars 25 μm, magnifications, 400×) and CCK-8 assays (B). **(C)** The cell cycle changes of MNNG/HOS cells after down-regulation of MALAT1 were determined by cell cycle analysis.

Also, as shown in the fore-mentioned section, there was a close relation between elevated MALAT1 and tumor size in osteosarcoma patients. Hence, we wondered about how the function MALAT1 might work on proliferation and cell cycle in MNNG/HOS cells. 5-ethynyl-2’-deoxyuridine (EdU) incorporation assays and CCK-8 assays as well as flow cytometry analysis were used to detect the proliferation ability and cell cycle changes, respectively. As the results displayed in Figure 3A, 3B, down-regulation of MALAT1 resulted in significant inhibition of cell proliferation. Meanwhile, a decrease of MALAT1 led to a significant accumulation of cells in the G0/G1 phase and a remarkable decrease of cells in the S phase (Figure 3C).

### MALAT1 was a target of miR-144-3p

**Figure 4:**
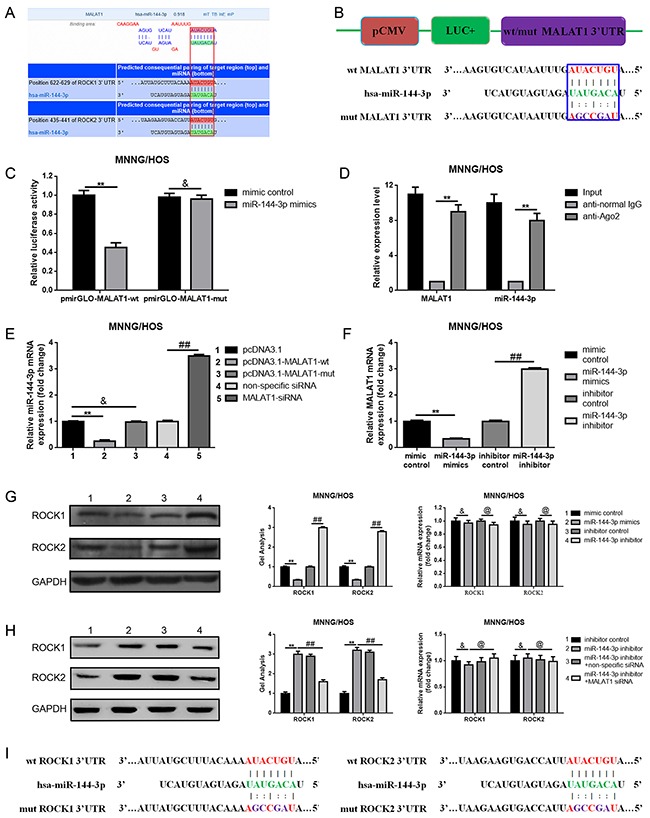

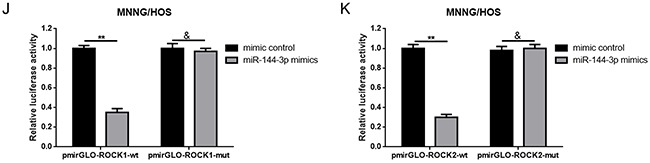
MALAT1 was a target of miR-144-3p **(A)** MALAT1 and ROCK1/ROCK2 shared the same theoretical miR-144-3p binding sites predicted by targetscan and DIANA-LncBase. **(B)** Diagram of the constructed MALAT1 reporter plasmid containing wild-type or mutant miR-144-3p binding sites. **(C)** Weakened fluorescence was present in miR-144-3p mimics and pmirGLO-MALAT1-wt co-transfection group as determined by luciferase assays. &P > 0.05, **P < 0.01 vs. mimic control group. **(D)** RIP assay was performed using input from cell lysate, normal mouse IgG or anti-Ago2. Relative expression levels of MALAT1 and miR-144-3p were determined by qRT-PCR. **P < 0.01 vs anti-normal IgG group. **(E)** Wild MALAT1 negatively regulated miR-144-3p expression measured by qRT-PCR. &P > 0.05, **P < 0.01 vs. pcDNA3.1 group, ##P < 0.01 vs. non-specific siRNA group. **(F)** miR-144-3p suppressed MALAT1 expression measured by qRT-PCR. **P < 0.01 vs. mimic control group, ##P < 0.01 vs. inhibitor control group. **(G)** ROCK1 and ROCK2 expression after up and down of miR-144-3p determined by western blot and qRT-PCR. &P > 0.05, **P < 0.01 vs. mimic control group, @P > 0.05, ##P < 0.01 vs. inhibitor control group. **(H)** Inhibition of miR-144-3p resulted in an elevation of ROCK1/ROCK2 and the elevating effect was reversed in the absence of MALAT1. &P > 0.05, @P > 0.05, **P < 0.01, ##P < 0.01 vs. miR-144-3p inhibitor group. **(I)** Diagram of the constructed ROCK1 and ROCK2 reporter plasmid containing wild-type or mutant miR-144-3p binding sites. **(J** and **K)** Weakened fluorescence was present in miR-144-3p mimics and pmirGLO-ROCK1-wt/pmirGLO-ROCK2-wt co-transfection group as determined by luciferase assays. &P > 0.05, **P < 0.01 vs. mimic control group. Data are shown as mean ± SD from three independent experiments.

It is widely reported that one of the mechanisms that show how lncRNAs are working is that lncRNAs could regulate their target genes by acting as a “sponge” or competing endogenous RNAs (ceRNA) of some miRNAs. Previous study has demonstrated that miR-144 could suppress osteosarcoma growth and metastasis by targeting ROCK1 and ROCK2 [20, 24], so we wondered whether there was a similar “ceRNA” network among MALAT1, ROCK1/ROCK2 and miR-144-3p. Firstly, through the online bioinformatics prediction software, Targetscan and DIANA-LncBase we found that MALAT1 and ROCK1/ROCK2 shared the same theoretical miR-144-3p binding sites (Figure 4A). Secondly, we constructed the luciferase reporter plasmids pmirGLO-MALAT1-wt and pmirGLO-MALAT1-mut which contained a wild type or mutant miR-144-3p putative binding sites in MALAT1, respectively (Figure 4B). The following dual luciferase assay showed that a significant weakening of fluorescence was presented in pmirGLO-MALAT1-wt and miR-144-3p mimics co-transfection group but not in pmirGLO-MALAT1-mut and miR-144-3p mimics co-transfection group (Figure 4C). Meanwhile, a RNA-binding protein immunoprecipitation (RIP) assay was performed to determine whether MALAT1 and miR-144-3p were in the same RNA induced silencing complex (RISC). The outcome in Figure 4D indicated that the level of MALAT1 and miR-144-3p was higher in anti-Ago2 group than that in anti-normal IgG group. Thirdly, we found a reciprocal inhibitory effect between MALAT1 and miR-144-3p, while interestingly, our findings also displayed that pcDNA3.1-MALAT-wt but not pcDNA3.1-MALAT-mut could repress miR-144-3p expression (Figure 4E, 4F). Fourthly, we verified that miR-144-3p could negatively regulate ROCK1/ROCK2 at the post-transcriptional level (Figure 4G). Simultaneously, as the results show in Figure 4H, we found that depression of miR-144-3p led to an elevation of ROCK1/ROCK2 protein, but the elevating effect was reversed in the absence of MALAT1 (co-transfection of miR-144-3p inhibitor and MALAT1 siRNA). Finally, through a luciferase assay we confirmed that miR-144-3p could bind to ROCK1/ROCK2 3’ untranslated regions (3’UTR) (Figure 4I-4K).

### MALAT1 promoted ROCK1/ROCK2 expression and their mediated proliferation and metastasis in a ceRNA manner via miR-144-3p

**Figure 5:**
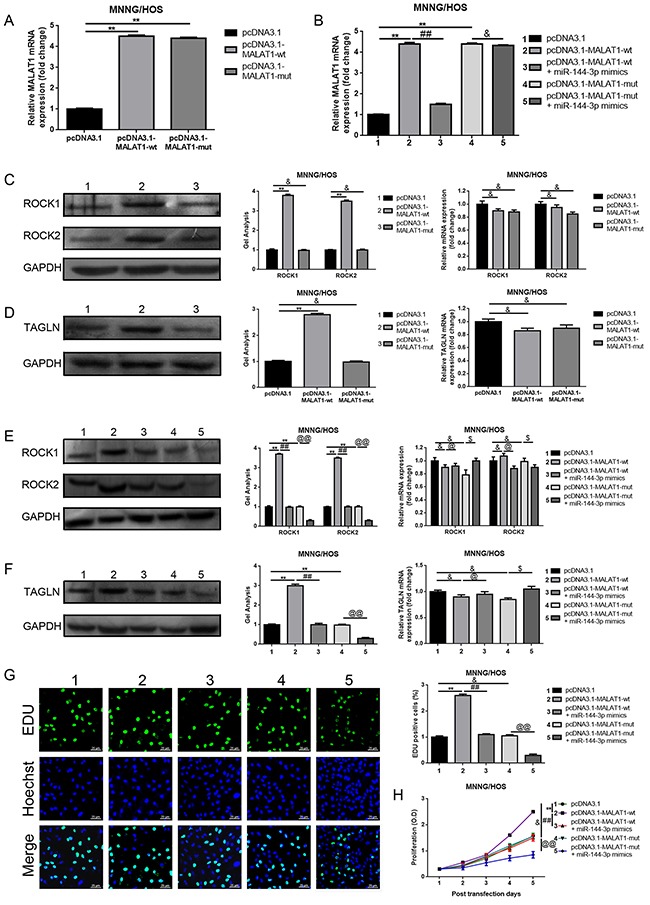

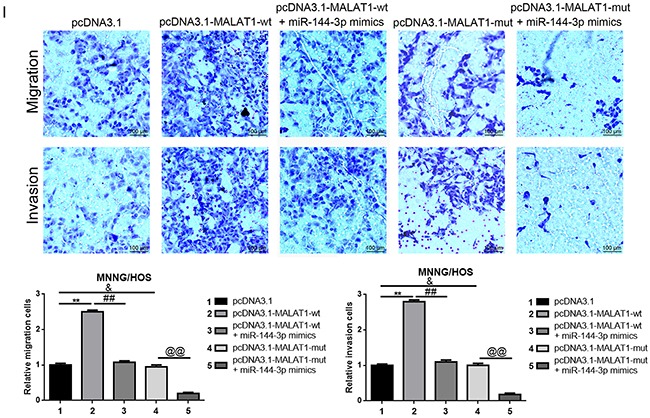
MALAT1 promoted ROCK1/ROCK2 expression and their mediated proliferation and metastasis in a ceRNA manner via miR-144-3p **(A)** Up-regulated MALAT1 after transfection of wild or mutant MALAT1 over-expression plasmids was confirmed by qRT-PCR. &P > 0.05, **P < 0.01 vs. pcDNA3.1 group. **(B)** The expression of MALAT1 after co-transfection of MALAT1 over-expression plasmids and miR-144-3p mimics was qualified by qRT-PCR. **P < 0.01 vs. pcDNA3.1 group, ##P < 0.01 vs. pcDNA3.1-MALAT1-wt group, &P > 0.05 vs. pcDNA3.1-MALAT1-mut group. **(C** and **D)** ROCK1/ROCK2 (C) and TAGLN (D) expression after transfection of wild or mutant MALAT1 over-expression plasmids were determined by western blot and qRT-PCR. &P > 0.05, **P < 0.01 vs. pcDNA3.1 group. **(E** and **F)** ROCK1/ROCK2 (E) and TAGLN (F) expression after co-transfection of MALAT1 over-expression plasmids and miR-144-3p mimics was qualified by western bolt and qRT-PCR. &P > 0.05, **P < 0.01 vs. pcDNA3.1 group, @P > 0.05, ##P < 0.01 vs. pcDNA3.1-MALAT-wt group, $P > 0.05, @@P < 0.01 vs. pcDNA3.1-MALAT-mut group. **(G** and **H)** The proliferation ability with respect to changes of MNNG/HOS after co-transfection of MALAT1 over-expression plasmids and miR-144-3p mimics were qualified by EDU (G, Scale bars 25 μm, magnifications, 400×) and CCK-8 assays (H). &P > 0.05, **P < 0.01 vs. pcDNA3.1 group, ##P < 0.01 vs. pcDNA3.1-MALAT1-wt group, @@P > 0.05 vs. pcDNA3.1-MALAT1-mut group. **(I)** The metastatic ability changes of MNNG/HOS after co-transfection of MALAT1 over-expression plasmids and miR-144-3p mimics were qualified by transwell assays (scale bars 100 μm, magnifications, 10×). &P > 0.05, **P < 0.01 vs. pcDNA3.1 group, ##P < 0.01 vs. pcDNA3.1-MALAT1-wt group, @@P > 0.05 vs. pcDNA3.1-MALAT1-mut group.

In the above sections, we verified that MALAT1, ROCK1 and ROCK2 were the targets of miR-144-3p and that decreased MALAT1 led to a significant reduction of ROCK1/ROCK2 but an elevation of miR-144-3p. Hence, we wondered whether MALAT1 affected ROCK1/ROCK2 via a ceRNA of miR-144-3p. We firstly confirmed that transfection of pcDNA3.1-MALAT1-wt resulted in an increase of ROCK1/ROCK2 protein expression but with normal mRNA expression. However, when the theoretical binding sites for miR-144-3p in MALAT1 were mutated (transfection of pcDNA3.1-MALAT1-mut), the facilitative effect MALAT1 worked on with regard to the ROCK1/ROCK2 protein was dismissed (Figure 5A, 5C). Meanwhile, the expression of TAGLN [25] - another reported downstream target of miR-144, also presented the same tendency as ROCK1/ROCK2 (Figure 5D). All the findings shown above indicate that MALAT1 regulated ROCK1/ROCK2 via the miR-144-3p pathway. Furthermore, miR-144-3p mimics were applied to further confirm the ceRNA mechanism among MALAT1, ROCK1/ROCK2 and miR-144-3p. As displayed in Figure 5B, the expression level of MALAT1 was elevated by pcDNA3.1-MALAT1-wt and pcDNA3.1-MALAT1- mut, but when miR-144-3p was involved in, the facilitative effect of MALAT1 over-expression plasmids displayed were reversed in pcDNA3.1-MALAT1-wt and miR-144-3p mimics co-transfection group but not in pcDNA3.1-MALAT1-mut and miR-144-3p mimics co-transfection group. Even further, the expression of ROCK1/ROCK2 and TAGLN presented the same tendency post-transcriptionally as MALAT1 (Figure 5E, 5F). These outcomes convincingly confirmed that MALAT1 affected ROCK1/ROCK2 via a ceRNA of miR-144-3p. Lastly, the incorporation assays as well as CCK-8 assays and transwell assays were re-executed to finally determine whether MALAT1 could regulate ROCK1/ROCK2 and their mediated proliferation/metastasis via a ceRNA of miR-144-3p in osteosarcoma cells MNNG/HOS. As shown in Figure 5G-5I, transfection of pcDNA3.1-MALAT1-wt resulted in a facilitation on proliferation/metastasis, but when the theoretical binding sites for miR-144-3p in MALAT1 was mutated (transfection of pcDNA3.1-MALAT1-mut), the facilitative effect was dismissed. Also, the facilitative effect pcDNA3.1-MALAT1-wt played on proliferation/metastasis could be reversed by miR-144-3p (transfection of miR-144-3p mimics). In brief, all the data above confirmed that MALAT1 promoted proliferation/metastasis by promoting ROCK1/ROCK2 via a ceRNA of miR-144-3p in osteosarcoma cells MNNG/HOS.

### Up-regulation of MALAT1 promoted tumorigenesis of osteosarcoma *in vivo*

**Figure 6:**
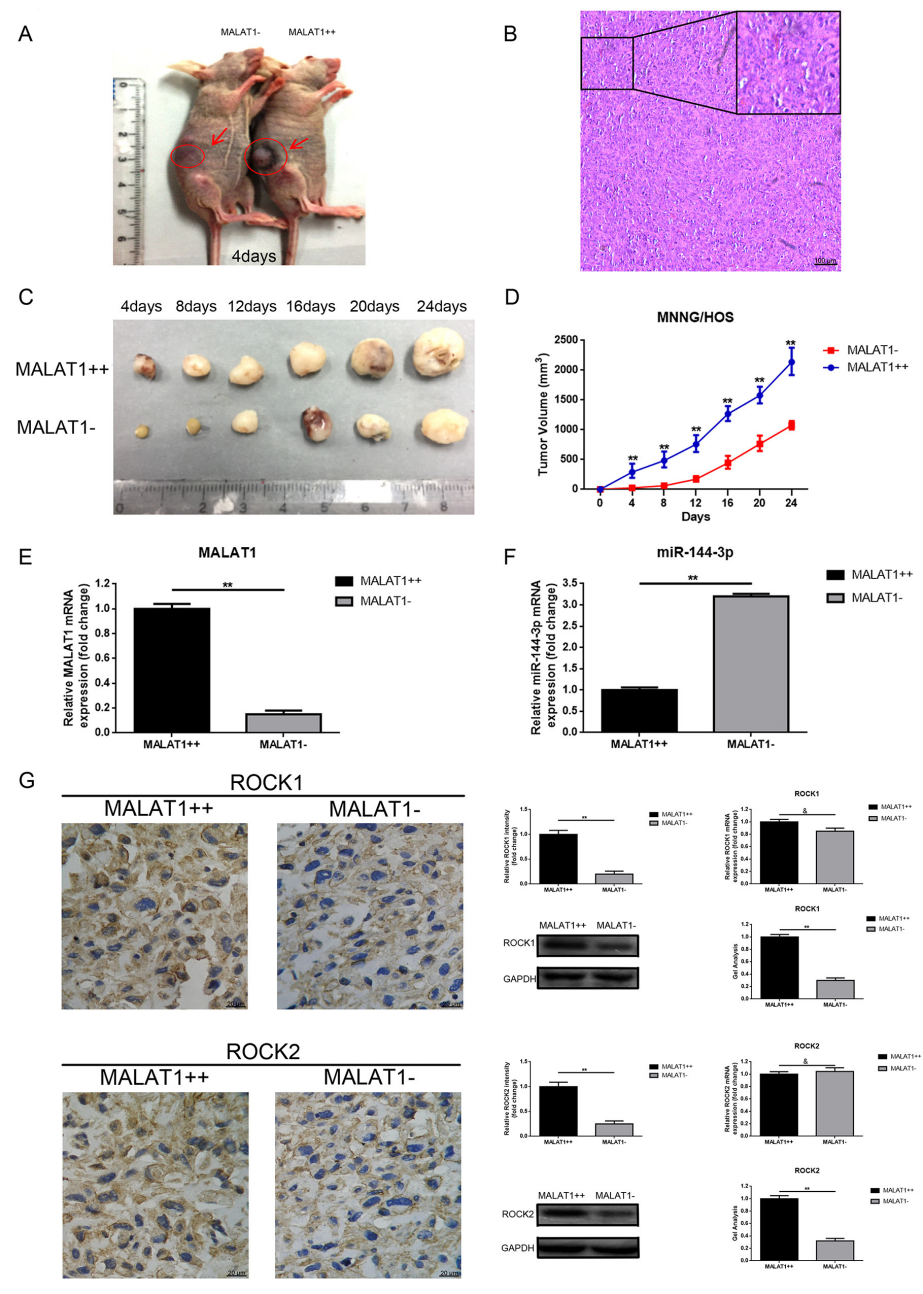
Up-regulation of MALAT1 promoted tumorigenesis of osteosarcoma *in vivo* **(A)** Representative photos of tumor formation in nude mice at the 4th day after inoculation as shown. **(B)** Representative image of H&E staining of excision formatted tumor tissues was shown (scale bars 100 μm, magnifications, 10× and 40×). **(C)** Representative photos of formatted tumors are shown. **(D)** Growth curve of tumor volumes. **(E)** MALAT1 expression in the formatted tumors was qualified by qRT-PCR. **(F)** MiR-144-3p expression in the formatted tumors was also qualified by qRT-PCR. **(G)** ROCK1 and ROCK2 expression in the formatted tumors were qualified by IHC (scale bars 20 μm, magnifications, 400×), western blot, qRT-PCR, respectively. **P < 0.01, &P > 0.05 vs. MALAT1++ group.

To explore the relationship between MALAT1 and tumorigenesis *in vivo*, MNNG/HOS cells with stable overexpression of MALAT1 (MALAT1++) and with corresponding negative control (MALAT1-) were subcutaneously inoculated into nude mice. As presented in Figure 6A, tumor volume in the MALAT1 overexpression (MALAT1++) group was visibly greater than that found in the negative control (MALAT1-) group at the 4th day after inoculation. Moreover, tumor growth in MALAT1++ group was markedly faster than that in MALAT1- group in the following 24 days after injection (Figure 6C, 6D). Even further, we detected MALAT1, miR-144-3p and ROCK1/ROCK2 expression in the formatted subcutaneous tumor nude mice specimens. As shown in Figure 6E, 6F, compared to MALAT1- group, MALAT1 expression was even higher in the MALAT1++ group, while the expression of miR-144-3p presented a contrary tendency. Meanwhile, elevated ROCK1/ROCK2 was displayed in the MALAT1++ group compared to that in the MALAT1- group in protein level while the difference in mRNA level was not significant (Figure 6G). These results demonstrated that MALAT1 promoted the tumorigenesis of osteosarcoma *in vivo*.

### Up-regulation of MALAT1 promoted pulmonary metastasis of osteosarcoma *in vivo*

**Figure 7:**
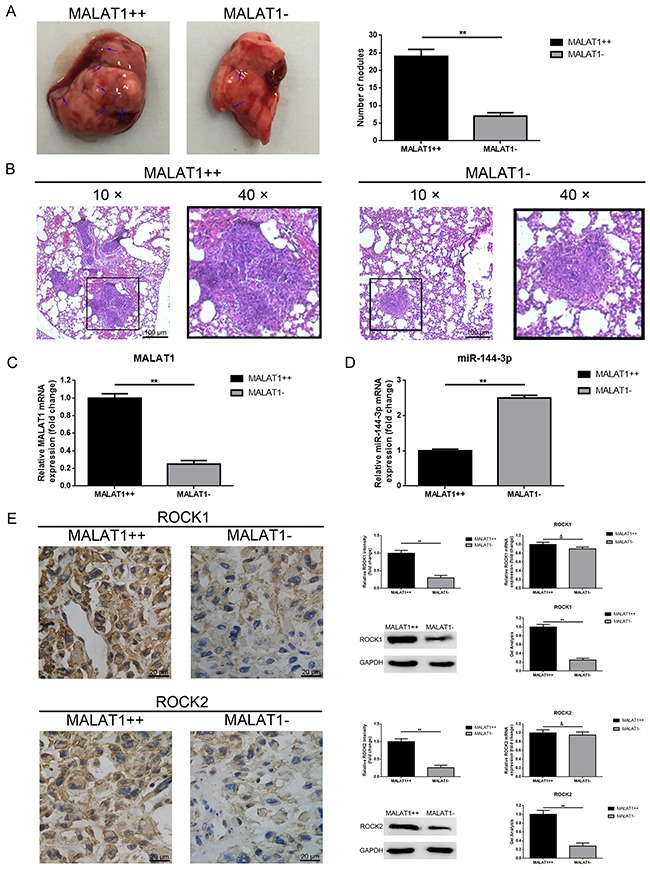
Up-regulation of MALAT1 promoted pulmonary metastasis of osteosarcoma *in vivo* **(A)** Representative photos of the metastatic nodules in the lungs were shown. **(B)** H&E staining of the lung sections with metastatic nodules were shown (scale bars 100 μm, magnifications, 10× and 40×). **(C)** MALAT1 expression in the metastatic nodules was qualified by qRT-PCR. **(D)** MiR-144-3p expression in the metastatic nodules was also qualified by qRT-PCR. **(E)** ROCK1 and ROCK2 expression in the metastatic nodules were qualified by IHC (Scale bars 20 μm, magnifications, 400×), western blot, qRT-PCR, respectively. **P < 0.01, &P > 0.05 vs. MALAT1++ group.

To determine the function of how the MALAT1 worked on osteosarcoma metastasis *in vivo*, the lungs of nude mice in group (n=6) were harvested at 6 weeks post inoculation. As the representative photos and HE staining as displayed in Figure 7A, 7B, the number of tumor nodules in total lungs of the MALAT1++ group were significantly more than that of the MALAT1- group, indicating that MALAT1 significantly promoted pulmonary metastasis in osteosarcoma. Meanwhile, we measured the expression of MALAT1 and miR-144-3p in the harvested nodules by using qRT-PCR. As the data presented in Figure 7C, 7D, the expression of MALAT1 in the MALAT1++ group was visibly higher than that of the MALAT1- group, but the expression of miR-144-3p presented a contrary tendency. Furthermore, we detected ROCK1/ROCK2 expression in the harvested nodules by using IHC, western blot and qRT-PCR. As shown in Figure 7E, elevated ROCK1/ROCK2 was present in the MALAT1++ group similar to the MALAT1- group with respect to protein level but a difference in mRNA level that was not significant. All these findings confirmed that up-regulation of MALAT1 promoted pulmonary metastasis of osteosarcoma via the miR-144-3p/ROCK1/ROCK2 pathway *in vivo*.

## DISCUSSION

Currently, more and more evidence has shown that non-coding RNAs play key roles in epigenetic regulation [26]. Non-coding RNAs are generally divided into miRNAs and lncRNAs according to length, and lncRNAs are a class of crucial and popular non-coding RNAs that presently contain more than 200 nucleotides in length [27]. It is widely reported that lncRNAs involved in multiple malignant tumors including osteosarcoma [28–32]. MALAT1 is located at chromosome 11q13.1 and was discovered as a metastatic prognostic marker for lung cancer [11]. Also, MALAT1 has been linked to several other human tumor entities including osteosarcoma [16, 33–38]. Recently, Dong found that MALAT1 could activate the PI3K/Akt pathway to promote cell proliferation, invasion and metastasis of osteosarcoma [39]. Liu K reported that MALAT1 promoted cell growth but inhibited apoptosis by regulation of HMGB1 via miR-142-3p and miR-129-5p [40]. In the present study, we found MALAT1 was overexpressed in osteosarcoma tissues and cell lines, and the increased MALAT1 was closely correlated with clinical stage, distant metastasis and tumor size in clinical patients, indicating that MALAT1 was an oncogene in tumorigenesis and distal metastasis of osteosarcoma.

ROCK1 and ROCK2 are the small molecule G proteins, which belong to the Rho (Rashomologue) family [41, 42]. ROCK1 and ROCK2 could regulate cell division and proliferation, regulate cytoskeletal activities and thereby affect cell movement [43–45]. Also, ROCK1 and ROCK2 could affect the angiogenesis, pericellular matrix degradation through their downstream effector molecules. Previous studies including ours reported that ROCK1 and ROCK2 were closely involved in osteosarcoma proliferation and migration/invasion [20, 23, 46]. The expression of ROCK1/ROCK2 was regulated by various upstream molecules including microRNAs (miRNAs) [47–51].

MiRNAs, 20-200 nucleotides in length, are another series of non-coding RNAs that regulate gene expression at the post-transcriptional/translational level. As a member of the miRNAs, miR-144-3p is widely reported as a pivotal molecule in various cell processes, such as cell differentiation, proliferation, apoptosis, migration and invasion [52–56] especially in tumors. The target genes of miR-144-3p include pre-leukemia transcription factor 3 (PBX3) in gastric cancer, zinc finger E-box-binding homeobox 1 and 2 (ZEB1 and ZEB2) in thyroid cancer, mitogen-activated protein kinase 8 (MAP3K8) in renal carcinoma, glucose transporter 1 (GLUT1) in lung cancer and ROCK1/ROCK2 and TAGLN in osteosarcoma [20, 25, 54, 57–60]. According to the outcomes of the present study, we found that up- and down-regulation of miR-144-3p could negatively affect ROCK1/ROCK2 expression at the post-transcriptional level, and the luciferase assays verified that ROCK1/ROCK2 was a target of miR-144-4p. Also, we revealed that MALAT1 was a target of miR-144-3p. Importantly, we found that different expression levels of MALAT1 could simultaneously regulate ROCK1/ROCK2 and TAGLN expression, and this phenomenon convincingly shows that MALAT1 affects ROCK1/ROCK2 via the miR-144-3p pathway.

A competing endogenous RNA (ceRNA) theory was first proposed by Leonardo Salmena in 2011 and is extensively studied and reported [61]. A brief understanding of ceRNA is that lncRNA, mRNA transcripts and false gene transcripts can affect each other by competitively combining with a miRNA response element (MRE) to influence post-transcriptional regulation. In the present study, we found that MALAT1 and ROCK1/ROCK2 shared the same miR-144-3p binding sites. Furthermore, we affirmed that there was a reciprocal repression effect between MALAT1 and miR-144-3p. More convincingly, we verified that only the wild-type MALAT1 overexpression plasmid could affect ROCK1/ROCK2 expression and change their mediated biological behaviors – proliferation and metastasis. When the theoretical miR-144-3p binding sites in MALAT1 were mutated, the facilitative effect of MALAT1 on ROCK1/ROCK2 was dismissed. Furthermore, the executed antisense experiments demonstrated that miR-144-3p mimics could reverse the facilitative effect MALAT1 had on ROCK1/ROCK2. These outcomes certified that MALAT1 regulated ROCK1/ROCK2 expression and their mediated proliferation/metastasis via a manner found in ceRNA of miR-144-3p. Lastly, through the vivo experiment of nude mice, we also found that the transfection of MALAT1 shRNAs slowing down the rate of tumor formation *in vivo*, inhibited lung metastasis of osteosarcoma cells. IHC, Western blot and RT-PCR showed the same results with the *in vitro* trend.

In conclusion, the present study demonstrated that MALAT1 was elevated in osteosarcoma. Inhibition of MALAT1 decreased metastasis and proliferation by regulation of ROCK1/ROCK2 via ceRNA of miR-144-3p in osteosarcoma cells *in vitro*. Up-regulation of MALAT1 promoted tumorigenesis and distal metastasis in nude mice models. Our results indicate that MALAT1 could be a potential therapeutic target in molecular targeting treatment of osteosarcoma.

## MATERIALS AND METHODS

### Patients and tissue samples

Osteosarcoma tissues and paired para-tumor bone tissues (n=45) used in this study were collected with the permission of patients during tumorectomy in Central Hospital affiliated with the Shenyang Medical College and Liaoning Cancer Hospital & Institute between February 2012 and December 2016. None of the patients had received chemotherapy before surgery and all the 45 cases were diagnosed according to a definite pathological diagnosis and the clinical stages of these patients were determined according to the tumor node metastasis (TNM) classification of the International Union Against Cancer (UICC). The Institute Research Medical Ethics Committee of Central Hospital affiliated with the Shenyang Medical College and Liaoning Cancer Hospital & Institute granted approval of this study.

### Cell culture

Human osteoblast cell line hFOB 1.19 and human osteosarcoma cell lines MG-63, U2OS, MNNG/HOS were purchased from the Chinese Academy of Sciences Cell Bank (Shanghai, China) and were maintained in DMEM/F12 (Gibco, USA), DMEM (Gibco, USA), DMEM and MEM (Gibco, USA), respectively. All mediums were supplemented with 10 % (v/v) fetal bovine serum (FBS, Sigma, USA), 100 IU/ml penicillin (Baomanbio, China) and 100 mg/ ml streptomycin (Baomanbio, China). All osteosarcoma cell lines were cultured at 37°C while hFOB 1.19 was cultured at 34°C in a humidified atmosphere containing 5 % CO_2_.

### Plasmids construction

MALAT1 fragments containing miR-144-3p binding sites were amplified and cloned into pmirGLO vectors (Promega, USA) to gain the reporter plasmid pmiRGLO- MALAT1-wild-type (pmirGLO- MALAT1-wt). The putative binding site of miR-144-3p in MALAT1 was mutated by using a QuikChange Site-Directed Mutagenesis kit (Agilent, USA) to synthetize pmirGLO-MALAT1-mutant-type (pmirGLO-MALAT1-mut). The reporter plasmids of pmirGLO-ROCK1-wt/pmirGLO-ROCK2-wt and pmirGLO-ROCK1-mut/pmirGLO-ROCK2-mut were constructed by using the same method. The above plasmids were used for the following luciferase reporter assays. Similarly, MALAT1 fragments containing miR-144-3p binding sites was amplified and cloned into the KpnI and XhoI restriction sites (Promega, USA) of pcDNA3.1 vector to synthetize pcDNA3.1-MALAT1-wild-type (pcDNA3.1-MALAT1-wt) and pcDNA3.1-MALAT1-mutant-type (pcDNA3.1-MALAT1-mut) was also gained by using QuikChange Site-Directed Mutagenesis kit (Agilent, USA). These two plasmids were used to construct MALAT1 over-expression cell models.

### Reverse transcription and quantitative real-time PCR

The procedure was carried out as previously described [62]. Total RNA or miRNA from osteosarcoma tissues and cells was extracted using TRIzol (Invitrogen, USA) according to the manufacturer's protocol. cDNA was synthesized from total RNA using a PrimeScript RT reagent Kit (TaKaRa, Dalian, China). PCR reactions containing SYBR Premix Ex Taq II (TaKaRa, Dalian, China) were next carried out with the following previously ascribed specific primers [20, 22]: ROCK1 forward 5’-AGGAAGGCGGACATATTAGTCCCT-3’, reverse 5’-AGACGATAGTTGGGTC CCGGC-3’; ROCK2 forward 5’-TTACATTGCTATCCACAGAACGG-3’, reverse 5’- CTATGCTGCTGCTTTTTGCTC-3’; TAGLN forward 5’-AATGGCGTGATTCTGAGCAA-3’, reverse 5’- CGATGCCTGCTGGAGCCGTCTA-3’; β-actin forward 5’-AGTGTGACGTGGACATCCGCAAAG-3’, reverse 5’-ATCCACATCTGCTGGAAGGTGGAC-3’; miR-144-3p forward 5’-GTCAAGAGCAATAACGAAAAATG-3’, reverse; 5’-GAGGTCAGGAGCAATAATGAA-3’; MALAT1 forward 5’-GCGACGAGTTGTGCTGCTATCT-3’, reverse 5’- ACACTGCTCTGGGTCTGCTTTT-3’. All reactions were performed in triplicate.

### Cell transfection and stable MALAT1 intervened cell lines

The procedure was carried out as previously described [63]. MNNG/HOS cells at 60~80% confluence were then selected for cell transfection. Plasmids were transfected into osteosarcoma cells by using Lipofectamine 2000 (Invitrogen, USA) according to the manufacturer's protocol. The sequence of MALAT1 siRNAs were as follows as ascribed before [21, 64]: 1# MALAT1 siRNA, 5’-GGCAAUGUUUUACACUAUUTT-3’; 2# MALAT1 siRNA, 5’-CACAGGGAAAGCGAGTGGTTGGTAA-3’; 3# MALAT1 siRNA, 5’-CACAGGGAAAGCGAGUGGUUGGU-3’; non-specific siRNA 5’-UUCUCCGAACGUGUCACGUTT-3’. MALAT1-siRNA and non-specific siRNA were purchased from Invitrogen (USA). To construct MALAT1 stable over-expression cell lines for further animal study, the stable transfected pcDNA3.1-MALAT1-wt and corresponding control (pcDNA3.1) cells were selected by the culture medium containing 0.4 mg/ml Geneticin (G418, Invitrogen). After 6 weeks, G418-resistant cell clones were established to construct MALAT1 stable over-expression cell lines.

### Cell proliferation assays

Cell counting Kit-8 (CCK-8) assay and 5-Ethynyl-20-Deoxyuridine (EDU) incorporation assay were used to evaluate MNNG/HOS cells’ proliferation abilities. For the CCK-8 assay, as previously reported [22], MNNG/HOS cells were seeded in 96-well plates (2 × 10^3^) supplemented with complete growth medium and followed by different transfection 24 h later. At days 1, 2, 3, 4 and 5 after transfection, 10μl CCK-8 solution was added into each well and incubated for 2 h. The absorbance was measured at an optical density of 450 nm using a Microplate reader (Bio-Rad, Hercules, CA). Experiments were repeated in triplicate. For EDU incorporation assay, EDU detection kits (Ribobio, China) were applied and the detailed processes were performed according to the manufacturer's instructions.

### Cell cycle analysis

MNNG/HOS cells were inoculated in 6-well plates with 1×10^6^ /L cells per well and incubated for 24h, then different kinds of management of the respective plasmids was executed. 24h later, the cells were harvested and washed twice by PBS, digested by trypsin (Santa Cruz, USA) and dissociated into single cell suspension. After that the cells were fixed by 70% acetic acid ethanol for 1h at 4°C, re-suspended in 100 ul propidium iodide (PI; Sigma, USA) solution and incubated for 30 min in the dark. FACS Calibur (BD, USA) was used to analyze the cell cycle.

### Western blotting

All the procedures were carried out as described [23]. Radio immunoprecipitation assay (RIPA) lysis buffer (Sigma, USA) was used to split cells. Protein samples were subjected to a 10 % SDS-PAGE and transferred onto a PVDF membrane, then blocked for 1h at room temperature. Each membrane was incubated with primary antibodies at 4 °C overnight and then secondary antibodies at room temperature for 1 h the next day. The following antibodies were used: mouse anti-ROCK1 antibody (1:1000, Cell signaling technology, USA), mouse anti-ROCK2 antibody (1:1000, Cell signaling technology, USA), mouse anti anti-TAGLN antibody (1:1000, Abcam, UK).

### Immunohistochemistry and evaluation

The procedure was performed as previously reported [65]. All slices were independently assessed by two experienced pathologists who were kept from knowing the patients’ clinical pathology and other information in order to ensure objective analysis. ROCK1 and ROCK2 expression level was evaluated via positive staining proportion methods and staining intensity of the tumor tissue. The immunoreactivity intensity was scored according to four values: 0) Negative staining; i) weak positive staining; ii) moderate positive staining; and iii) strong positive staining. Slices with inconsistent results were re-examined by the original two pathologists and a senior pathologist in order to come to a consensus. Sections were immunostained with mouse anti-ROCK1 and ROCK2 antibody (1:1000, Cell signaling technology, USA).

### Dual luciferase reporter assay

The procedure was carried out as previously described [23]. MiR-144-3p mimics/mimic control and the constructed reporter plasmids (pmirGLO-ROCK1-wt/pmirGLO-ROCK1-mut, pmirGLO-ROCK2-wt/pmirGLO-ROCK2-mut and pmirGLO-MALAT1-wt/pmirGLO-MALAT1-mut) were co-transfected into cultured MNNG/HOS cells by using Lipofectamine 2000 (Invitrogen, USA) and incubated for 48 h, respectively. Then the Dual-Luciferase Reporter Assay System (Promega, USA) was used to evaluate fluorescence intensity changes according to the manufacturer's protocol.

### RIP assay

The procedure using RIP was carried out using a Magna RNA-binding protein immunoprecipitation kit (Millipore, MA, USA) as previously described [66]. In brief, whole-cell lysate was incubated with a RIP buffer containing magnetic beads conjugated with human anti-Ago2 antibody, or negative control normal mouse IgG. Samples were incubated with Proteinase K and then immunoprecipitated RNA was isolated. The RNA concentration was measured by a spectrophotometer (Thermo Scientific, Waltham, MA, USA) and the RNA quality was assessed using a bio-analyzer (Agilent, Santa Clara, CA, USA). Furthermore, purified RNAs were extracted and analyzed by quantitative real-time PCR to demonstrate the presence of the binding targets.

### Wound-healing assay

After transfection for 48 h, the sub-confluent cell monolayers were formed into three parallel lines with a P-200 pipette tip. The detached cells were washed off twice gently, and the medium was then replaced with 1% FBS complete medium. To visualize wound healing, images were taken at 0, 24, and 48 h. The percentage of wound closure (Original width–Width after cell migration/Original width) was calculated.

### Transwell assay

The procedures of transwell assays were performed as described before [23]. In brief, MNNG/HOS cells were seeded on uncoated and Matrigel-coated upper chambers (BD Bioscience, USA) for migration and invasion assays respectively. Medium without serum and with 10% FBS was supplemented into the upper and lower wells respectively for a further 24 h incubation. Afterwards, the non-migrating or non-invading cells were carefully wiped out. Then the filters were fixed in 90 % alcohol and followed by crystal violet stain. Five random fields were counted per chamber by using an inverted microscope (Olympus, Japan), and each experiment was repeated three times.

### Animal study

Female nude mice (4 - 5 weeks old) were purchased from Animal Care and Use Committee of Dalian Medical University Ltd., and kept under sterile specific pathogen-free conditions. MNNG/HOS osteosarcoma cells with stable overexpression of MALAT1 (MALAT1++) and with corresponding negative control (MALAT1-) were harvested from 6-well cell culture plates. About 1 ×10^6^ cells in 50% matrigel (BD Bioscience) were injected into the subcutaneous and caudal vein of the mice. In the subcutaneous group, mice were killed every 4 days for 24 days, tumor growth was measured, and tumor volume was calculated using the formula, volume=1/2 (length × width^2^). 7 weeks after injection, the mice given caudal vein injection were dissected to observe the pulmonary metastasis changes. All experimental procedures were carried out in compliance with the guiding principles for the Care and Use of Animals described in the American Journal of Physiology and with the Guidelines established by the Institute of Laboratory Animal Sciences, Faculty of Medicine, Kagoshima University. All efforts were made to minimize animal suffering, to reduce the number of animals used, and to utilize possible alternatives to *in vivo* techniques.

### Statistical analysis

All experiments were repeated in triplicate and all data from three independent experiments were expressed as a mean ± SD. GraphPad Prism V5.0 (GraphPad Software, Inc., USA) software and SPSS 19.0 statistical software were used for statistical analysis. Pearson's chi-squared test was used to analyze the correlation between MALAT1 and clinicopathological features. Meanwhile, a log-rank test was used for survival analysis by using a GraphPad Prism V5.0. Differences in the two groups were analyzed by using the Student's t-test or one-way ANOVA. Differences were considered significant if P < 0.05.
